# Nanofibrous nerve guidance conduits decorated with decellularized matrix hydrogel facilitate peripheral nerve injury repair

**DOI:** 10.7150/thno.50825

**Published:** 2021-01-01

**Authors:** Chushan Zheng, Zehong Yang, Shihao Chen, Fang Zhang, Zilong Rao, Cailing Zhao, Daping Quan, Ying Bai, Jun Shen

**Affiliations:** 1 Department of Radiology, Sun Yat-sen Memorial Hospital, Sun Yat-sen University, Guangzhou, Guangdong 510120, China.; 2 Guangdong Provincial Key Laboratory of Malignant Tumor Epigenetics and Gene Regulation, Medical Research Center, Sun Yat-sen Memorial Hospital, Sun Yat-sen University, Guangzhou, Guangdong 510120, China.; 3 PCFM Lab, GD HPPC Lab, School of Chemistry, Sun Yat-sen University, Guangzhou, Guangdong 510275, China.; 4 Guangdong Functional Biomaterials Engineering Technology Research Center, School of Materials Science and Engineering, Sun Yat-sen University, Guangzhou, Guangdong 510275, China.

**Keywords:** decellularized nerve matrix hydrogel, peripheral nerve injury, nerve guidance conduit, magnetic resonance imaging, electrospinning

## Abstract

**Rationale:** Peripheral nerve injury (PNI) is a great challenge for regenerative medicine. Nerve autograft is the gold standard for clinical PNI repair. Due to its significant drawbacks, artificial nerve guidance conduits (NGCs) have drawn much attention as replacement therapies. We developed a combinatorial NGC consisting of longitudinally aligned electrospun nanofibers and porcine decellularized nerve matrix hydrogel (pDNM gel). The in vivo capacity for facilitating nerve tissue regeneration and functional recovery was evaluated in a rat sciatic nerve defect model.

**Methods:** Poly (*L*-lactic acid) (PLLA) was electrospun into randomly oriented (PLLA-random) and longitudinally aligned (PLLA-aligned) nanofibers. PLLA-aligned were further coated with pDNM gel at concentrations of 0.25% (PLLA-aligned/0.25% pDNM gel) and 1% (PLLA-aligned/1% pDNM gel). Axonal extension and Schwann cells migration were evaluated by immunofluorescence staining of dorsal root ganglia cultured on the scaffolds. To fabricate implantable NGCs, the nanofibrous scaffolds were rolled and covered with an electrospun protection tube. The fabricated NGCs were then implanted into a 5 mm sciatic nerve defect model in adult male Sprague-Dawley rats. Nerves treated with NGCs were compared to contralateral uninjured nerves (control group), injured but untreated nerves (unstitched group), and autografted nerves. Nerve regeneration was monitored by an established set of assays, including T2 values and diffusion tensor imaging (DTI) derived from multiparametric magnetic resonance imaging (MRI), histological assessments, and immunostaining. Nerve functional recovery was evaluated by walking track analysis.

**Results:** PLLA-aligned/0.25% pDNM gel scaffold exhibited the best performance in facilitating directed axonal extension and Schwann cells migration in vitro due to the combined effects of the topological cues provided by the aligned nanofibers and the biochemical cues retained in the pDNM gel. Consistent results were obtained in animal experiments with the fabricated NGCs. Both the T2 and fractional anisotropy values of the PLLA-aligned/0.25% pDNM gel group were the closest to those of the autografted group, and returned to normal much faster than those of the other NGCs groups. Histological assessment indicated that the implanted PLLA-aligned/0.25% pDNM gel NGC resulted in the largest number of axons and the most extensive myelination among all fabricated NGCs. Further, the PLLA-aligned/0.25% pDNM gel group exhibited the highest sciatic nerve function index, which was comparable to that of the autografted group, at 8 weeks post-surgery.

**Conclusions:** NGCs composed of aligned PLLA nanofibers decorated with 0.25% pDNM gel provided both topological and biochemical guidance for directing and promoting axonal extension, nerve fiber myelination, and functional recovery. Moreover, T2-mapping and DTI metrics were found to be useful non-invasive monitoring techniques for PNI treatment.

## Introduction

Peripheral nerve injury (PNI) is a common clinical disease that leads to sensory and motor deficits. Neurotmesis, a complete transection or disruption of the entire nerve, is the most severe form of PNI. The long-distance defect/gap between the proximal and distal stumps of the damaged nerve often results in limited regeneration capacity 1, 2. Autologous nerve grafting is the gold standard for clinical treatment of peripheral nerve defects 3. However, the major drawbacks of nerve autografts, such as limited donor tissue, secondary body injury, repeated surgeries, and size/structural disproportion of grafted nerve tissue, are also significant 4, 5. Artificial nerve guidance conduits (NGCs) were invented for bridging the two stumps of the defected nerves 6. To date, a large variety of NGCs have been developed to overcome the disadvantages of nerve autografts and provide alternative therapeutic approaches for treatment of PNI 7. However, most of the FDA-approved NGCs that consist of either biodegradable synthetic polymers or natural biomaterials (e.g., collagen) are only fabricated as single tubular structures 8, 9. Generally, these conduits provide a large space for nerve fiber regeneration once sutured between the proximal and distal ends of the defect, but they also lead to axonal dispersion due to the lack of topological and biological guidance, which is inhibitory to nerve functional recovery 10, 11. Therefore, rational selection of functional materials and smart design of microscopic architectures are both essential in current advancements of NGCs 6, 12.

Within the last decade, tissue-engineered nerve grafts have been developed from biomaterial-based scaffolds combined with additional growth factors 13 and other bioactive molecules 14 to trigger neurogenesis and facilitate restoration of neural functions 15, 16. As a type of bioactive scaffold, decellularized extracellular matrix (dECM) derived from tissues or organs has attracted increasing attention due to its tissue specificity and preservation of the composition and microstructure of native extracellular matrix (ECM) 17, 18. dECM is often more than a scaffold, serving as a functional regulator of cell differentiation, signal transduction, proliferation, morphogenesis, and remodeling in the host 11. Furthermore, many tissue-derived dECMs undergo enzymatic digestion to form dECM hydrogels via sol-gel transition 19-21. For instance, decellularized nerve matrix hydrogel derived from porcine sciatic nerves (pDNM gel) was reported to effectively promote neurite remyelination but inhibit synaptogenesis in vitro 22.

Besides the bioactivity of the scaffolding material, major challenges in peripheral nerve regeneration involve misdirected axonal growth. It has been widely acknowledged that longitudinally aligned fiber-like or channel-like microstructures critically contribute to the guidance of neurite outgrowth 23-26. Pioneering studies have also shown that aligned electrospun nanofibers can support oriented axonal extension and glial cells (Schwann cells, SCs) migration from cultured primary dorsal root ganglion (DRG) explants 27, 28. Furthermore, in vitro studies suggest that longitudinally aligned nanofibers can accelerate neurite extension compared to nanofibers with random alignments 29-31. Our previous study showed clear evidence that aligned nanofibers coated with pDNM gel can facilitate synergistic effects in directed axonal extension and SC migration in seeded DRG explants 32. We demonstrated that pDNM gel-accelerated SC migration can further promote and guide neurite outgrowth along the aligned nanofibers. However, whether composite pDNM gel-coated nanofibrous scaffolds contribute to nerve regeneration and functional recovery in a PNI animal model remains to be evaluated.

Herein, longitudinally aligned poly (*L*-lactic acid) (PLLA) nanofibers were precoated with pDNM gel and fabricated into tissue-engineered NGCs with electrospun protection tubes. These prepared nanofiber NGCs were implanted into a defected sciatic nerve rat model. The performance of these implanted NGCs was evaluated in comparison with the contralateral uninjured side (control group), untreated injured nerves (unstitched group), as well as autografted nerves. Magnetic resonance imaging (MRI) is a powerful noninvasive diagnostic tool for assessing PNI and nerve tissue regeneration via direct visualization of peripheral nerve changes 31. Quantitative MRI parameters, including T2 values and metrics of diffusion tensor imaging (DTI), can sensitively detect the continuity of neurites and indicate the extent of nerve dysfunction/regeneration at various timepoints, such as immediately following acute PNI, during the recovery phase, and before regenerated axons reach their target 33-36. In this study, sciatic nerve reinnervation in rats was assessed by multiparametric MRI and evaluated using T2-mapping and DTI. It was hypothesized that the combinatorial pDNM gel and aligned nanofibers scaffold could provide both biochemical and topological guidance for promoting axonal extension and functional recovery after PNI. Additionally, PNI repair could be monitored non-invasively and in situ by MRI.

## Materials and Methods

### Animals and ethics

All interventions and animal care procedures were performed following the Guidelines and Policies for Animal Surgery at Sun Yat-sen University (Guangzhou, China) and were approved by the Institutional Animal Use and Care Committee. 144 healthy adult male Sprague-Dawley (SD) rats (250 ± 20 g) were obtained from the Animal Experiment Centre of Sun Yat-sen University. The rodents were kept under a 12 h light/dark cycle with free access to food and water. The rats were transported and kept more than 7 days in advance of surgery.

### Preparation of electrospun nanofibrous scaffolds

Poly (*L*-lactic acid) (PLLA; PURAC, Groningen, Holland) was dissolved in 2,2,2-trifluoroethanol (TFE) (Aladdin, Shanghai, China) to obtain a 7.5% (w/v) polymer solution, then transferred into a syringe with a 20G stainless steel needle. The polymer solution was ejected at 1 mL/h by a syringe pump (Tonli, Shenzhen, China). To collect PLLA nanofibers, a high voltage power supply (Tonli, Shenzhen, China) was connected to the needle and set to 15 kV. The randomly oriented nanofibers were collected on clean glass slides on top of a steel plate. The distance between the needle and the collection plate was 100 mm. To produce longitudinally aligned nanofibers, a roller wheel with pre-attached glass coverslips was used as the collector with a rotational speed of 3000 rpm. After 20 min of nanofiber collection, the samples were vacuum-dried for 24 h to remove residual solvent.

### Decellularized nerve matrix hydrogel coating

Decellularized matrix hydrogel derived from porcine sciatic nerves (pDNM gel) was prepared following our previously reported protocols 34, 37. Briefly, fresh sciatic nerves harvested from miniature pigs were extracted with 3.0% Triton X-100 and 4.0% sodium deoxycholate and rinsed with sterile water three times until completely decellularized. The decellularized pDNM was lyophilized, pulverized into a powder, then solubilized in acidic pepsin solution (0.01 M HCl) to 1% (w/v) pDNM and 10/1 (w/w) pDNM/pepsin at room temperature for 24 h. The digested solution was centrifuged at 20,000 rpm (Beckman Coulter, Brea, USA) for 30 min to remove all undissolved contents, lyophilized and then pulverized to powder. The powder was re-dissolved in 0.01 M HCl to 2% (w/v), the pH was adjusted to 7.5 by addition of 0.1 M NaOH, and 10× PBS was added to form pDNM-sol. The sol was diluted to 0.25% (w/v) and 1% (w/v) with 1× PBS.

The aligned PLLA nanofibrous films were pre-sterilized with 75% ethanol, immersed in 0.25% (w/v) or 1% (w/v) pDNM-sol for 10 min at 4 °C, then gelled at 37 °C for 20 min. The films were then lyophilized and stored at -40 °C before use. All solutions used were sterile, and all above-mentioned procedures were performed in a sterilized environment.

### Preparation of NGCs

Electrospun poly (*L*-lactic acid-co-trimethylene carbonate) P(LLA-TMC) nanofibers (LLA/TMC = 70/30) were used to fabricate the protection tubes of the implantable NGCs. PLLA-random, PLLA-aligned, PLLA-aligned/0.25% pDNM gel, or PLLA-aligned/1% pDNM gel scaffolds were first wrapped around a stainless steel rod (diameter = 2 mm) to serve as the collection substrates for electrospun P(LLA-TMC) nanofibers. P(LLA-TMC) tubes were fabricated from 15% (w/v) P(LLA-TMC) in TFE. The solution was ejected onto the collection substrates through a 0.65 mm needle charged at 15 kV and dispensed at a flow rate of 0.5 mL/h. The total length of each NGC was 10 mm.

### DRG culture and immunofluorescence staining

The scaffolds were immersed in 75% ethanol for 30 min and washed with PBS three times for sterilization. DRGs were dissected from newborn SD rats (postnatal day 1, supplied by the Laboratory Animal Center of Sun Yat-sen University, China). Residual nerve roots were cut off under a stereomicroscope. Then, the isolated DRGs were plated on PLLA nanofibrous films with or without pDNM-gel coating, placed in 48-well plates with neurobasal medium containing 2% B27, 0.3% L-glutamine, and 1% penicillin-streptomycin, and incubated at 37 ℃ in 5% CO_2_ and 92% humidity. The medium was changed every 2 days.

After culturing for 7 days, the samples were fixed in 4% paraformaldehyde in PBS for 20 min, rinsed with PBS, and permeabilized and blocked in 0.1% Triton X-100 and 10% donkey serum in PBS for 30 min. DRG samples were incubated with primary antibodies against NF200 (dilution 1/150) or S100 (dilution 1/1000) for 2 h at room temperature, followed by secondary antibodies conjugated to Fluro-488 (dilution 1/1000) or Fluro 594 (dilution 1/1000) for 1h. The samples were then rinsed with PBS for 10 min (3 times), stained with DAPI (dilution 1/2500) for 20 min, then observed by a laser scanning confocal microscope (Zeiss, Germany).

### Animal surgery and NGC implantation

Animals were randomly selected and placed into two groups. Each group was subdivided into six subgroups: autografted, unstitched, PLLA-random, PLLA-aligned, PLLA-aligned/0.25% pDNM gel, and PLLA-aligned/1% pDNM gel (n = 6 per subgroup in the first group, and n = 18 per subgroup in the second group). Following a previously reported procedure 35, 38, general anesthesia was administered by intraperitoneal injection of pentobarbital sodium (40 mg/kg) after isoflurane inhalation. Then, the animals were placed in a prone position. A 20 mm incision was made along the left femoral axis to separate the thigh muscles with the sciatic nerve dissected. The sciatic nerve was exposed, and its middle portion was removed to create a 5 mm gap between the proximal and distal stumps. For the autografted group, the transected 5 mm sciatic nerve was reversed and then grafted back using 8-0 sutures (Prolene; Jinhuan Medical Appliance, Shanghai, China) 39, 40. For the unstitched group, no further treatment was performed after the 5 mm sciatic nerve segment was excised. For the groups implanted with NGCs, the fabricated NGCs were implanted to bridge the proximal and distal ends of the nerve defects. Both nerve stumps were inserted into the tube lumen (depth = ~2 mm) and sutured to the conduit wall using a single epineurial 8-0 suture. The wounds were then closed, and the animals were returned to their cages 38.

The animals in the first group underwent MRI and sciatic nerve functional assessments at 1, 2, 6, and 8 weeks post-surgery. The rats in the second group were subjected to histologic analysis at 2, 6, and 8 weeks post-surgery. The uninjured nerve on the contralateral side of each animal was used as the control group.

### MRI acquisition and evaluation

MRI was performed on a 3.0 T scanner (Achieva; Philips Medical Systems, Best, Netherlands) with a 50 mm × 50 mm 4-channel phased-array rat coil (Chenguang Medical Technologies Co., Shanghai, China). After anesthesia, animals were placed in a supine position. Coronal fat-suppressed turbo spin-echo T2-weighted imaging (FS-T2WI), T2 mapping, and axial DTI data were acquired. Parameters of acquisition for FS-T2WI were as follows: repetition time (TR) = 1200 ms, echo time (TE) = 60 ms, acquisition matrix = 172 × 170, reconstruction matrix = 256 × 256, field of view (FOV) = 60 mm × 60 mm, number of signals averaged (NSA) = 1, and slice thickness/gap = 1 mm / 0 mm. T2 mapping was obtained using a multi-slice, multi-echo spin-echo sequence with the following parameters: TR = 1600 ms, TE = 20-160 ms, matrix = 148 × 171, FOV = 60 mm × 60 mm, NSA = 2, and slice thickness/gap = 2 mm / 0 mm. Axial DTI was obtained using an echoplanar imaging (EPI) sequence with the following acquisition parameters: TR = 1206 ms, TE = 79 ms, slice thickness/gap = 1.5 mm / 0.5 mm, b-value = 0.800 s/mm^2^, 16 gradients, FOV = 60 mm × 128 mm, NSA = 2, acquisition matrix = 76 × 56, voxel size = 1.6 mm, reconstruction matrix = 256 × 256, reconstruction voxel size = 0.188 mm, EPI factor = 7, and flip angle = 90°. The imaging plane was parallel to the longitudinal course of the sciatic nerve for coronal images and perpendicular to the sciatic nerve (covering the lesion epicenter and proximal and distal stumps) for DTI.

### Image analysis

Morphological abnormalities were assessed by FS-T2WI. Fractional anisotropy (FA) values were derived from DTI images of the distal stumps of injured nerves. T2 and FA values in regions of interest (ROI) were calculated from T2-mapping and DTI images on a workstation (View Forum; Philips) 38. Briefly, a rectangular ROI with a minimum size of 85 pixels was placed within the implanted NGCs. To reduce partial volume effects, images demonstrating the long axis of sciatic nerves were chosen and the ROI position was carefully adjusted to the middle of the NGCs and covering the nerve trunk. The ROIs for DTI metrics were manually traced in the implanted conduits on axial DWI images for quantitation of FA. To ensure accurate and consistent positioning of the ROIs, transverse DTI images were linked with coronal T2-weighted images. ROI was manually selected and placed on three adjacent levels, which covered the whole injured site or its contralateral side (length = ~6 mm). The measurements were performed independently and blindly by two radiologists. The diffusion tensor was calculated on a pixel-by-pixel basis to obtain the eigenvectors and values. FA values were derived for each voxel 40-42. Average values from the 2 datasets were used for analysis. Tractography was obtained using the fiber tracking analysis software available on the same workstation. A multiple ROI method was used to reconstruct diffusion tensor tractography (DTT) 43. Briefly, multiple ROIs were manually placed on each DTI axial image slice along the injured sciatic nerve from the proximal stump to the distal stump. Fibers passing through the multiple ROIs were visualized. The threshold of FA was set to 0.4, the maximum fiber angle was 27°, and the minimum fiber length was 15 mm 42.

### Histology

At 2, 6, and 8 weeks after surgery, 6 animals in each group were euthanized and transcardially perfused with PBS to remove red blood cells. For the groups implanted with NGCs and the autografted group, the distal portions, the nerve grafts along the same severed nerves, and the sham-operated nerves were harvested. For the unstitched group, only the distal portions and the sham-operated nerves were harvested. The morphologies of the implanted NGCs and the nerve connections inside the tubes were observed under an optical microscope. Then, each tissue sample was split into two parts from the center of the nerve and fixed in 4% paraformaldehyde. Contiguous 1 μm cross-sections were obtained and stained with toluidine blue 44, 45. The rest was longitudinally sectioned in 10 μm slices and immunofluorescence stained for small proline-rich protein 1A (SPRR1A) (Abcam, Cambridge, UK) to detect axonal regeneration 44. The sample slices were rinsed in PBS, incubated in 0.3% Triton X-100 (Abcam, Cambridge, UK) for 15 min, blocked with donkey serum (10:100 (v/v); Abcam) for 30 min, then incubated overnight at 4 °C with primary antibodies against SPRR1A (1:200 (v/v); Abcam). After rinsing another three times with PBS, slices were incubated with the corresponding Alexa Fluor 488-conjugated secondary antibodies (1:200 v/v; Invotrogen, Carlsbad, USA) at room temperature for 1 h and subsequently with DAPI for nuclei staining. The immunostained samples were observed using a laser scanning confocal microscope (LSM780; Carl Zeiss, Jena, Germany).

For quantification of toluidine blue staining, the entire cross-sectional area of the injured nerve (1360 × 1024 pixels, 15.5 pixels/μm) was observed using the 1000× oil lens of an optical microscope (Olympus BX63; Olympus, Tokyo, Japan). The center and distal parts of the nerve grafts were selected for imaging. Five different regions in each part of the injured nerve (area = 5797 μm^2^ per image, total area = 28,983 μm^2^) were randomly selected for analysis 35, 41. The number of axons, myelin thickness, and myelinated fiber diameter were measured using ImageJ (National Institutes of Health, Bethesda, Maryland).

### Functional recovery assessment

Walking track analysis was performed for the first group of animals at each time point prior to MRI to assess nerve functional recovery by two experimenters in consensus. Sciatic nerve function index (SFI), an indicator of locomotor nerve dysfunction, was scored using the following parameters: normal print length (NPL), normal toe spread (NTS), normal intermediary toe spread (NIT), experimental print length (EPL), experimental toe spread (ETS), and experimental intermediary toe spread (EIT). SFI was calculated using the following formula 41:

SFI = -38.3(EPL - NPL)/NPL + 109.5(ETS - NTS)/NTS + 13.3(EIT - NIT)/NIT - 8.8

### Statistical analysis

Repeated-measures one-way analysis of variance was used to evaluate the significance of T2, FA, and SFI values among the subgroups in the first group of animals, who underwent MRI and functional recovery assessments. Interobserver agreement and statistical power were also assessed. The T2, FA, and SFI values were compared between these subgroups using a Bonferroni test for multiple pairwise comparisons at each time point (1, 2, 6, and 8 weeks post-surgery) to determine the effect of pDNM gel concentration. Toluidine blue staining results were compared between subgroups in the second group of animals, who were subjected to histologic analysis, using one-way analysis of variance followed by a Bonferroni post-hoc test for multiple pairwise comparisons. Statistical analyses were performed using SPSS version 25.0 for Mac (SPSS, Inc., Chicago, Illinois). All data are presented as mean ± standard deviation. A 2-sided *p* < 0.05 was considered a significant difference.

## Results

### Fabrication of pDNM gel-decorated nanofibrous NGCs

**Figure 1:**
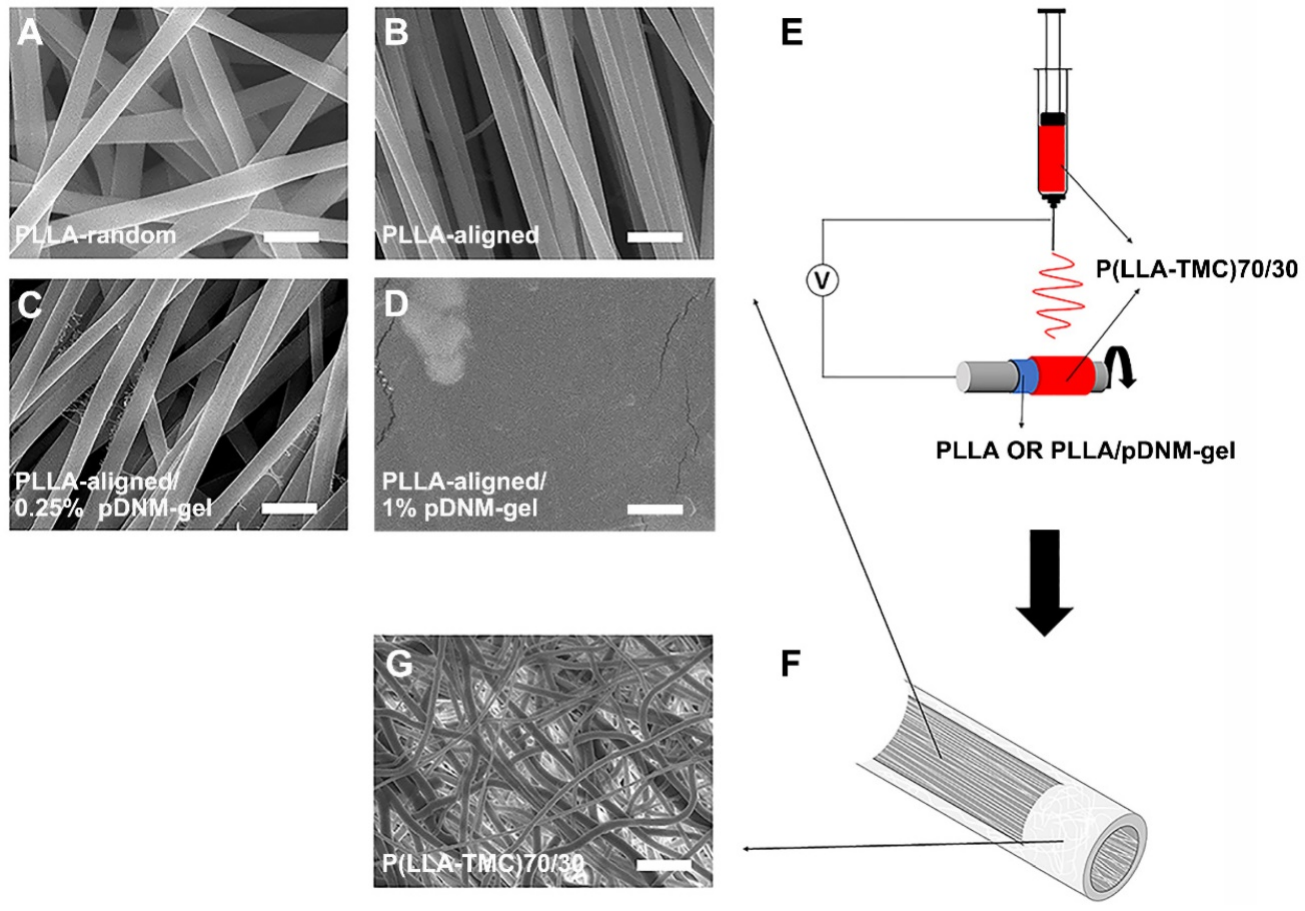
**pDNM gel-coated electrospun nanofibrous scaffolds and NGC fabrication.** Representative SEM micrographs of **(A)** randomly oriented electrospun PLLA nanofibers scaffolds (PLLA-random), **(B)** longitudinally aligned PLLA nanofibers scaffolds (PLLA-aligned), **(C)** PLLA-aligned scaffolds coated with 0.25% (w/v) pDNM gel (PLLA-aligned/0.25% pDNM gel), and **(D)** PLLA-aligned scaffolds coated with 1% (w/v) pDNM gel (PLLA-aligned/1% pDNM gel). Scale bars = 2 μm. **(E)** Schematic illustration of the fabrication process for P(LLA-TMC)70/30 protection tubes. The prepared nanofibrous PLLA scaffolds (with or without pDNM gel) were wrapped around a stainless-steel rod (diameter = 2 mm), then P(LLA-TMC)70/30 was electrospun onto and wrapped around the scaffolds. **(F)** Diagram of the fabricated NGCs illustrating the inner scaffold of electrospun PLLA nanofibers with or without pDNM gel coating and the outer protection tube consisting of dense P(LLA-TMC)70/30 nanofibers. **(G)** SEM image of P(LLA-TMC)70/30 nanofibers in the protection tubes. Scale bar = 10 μm.

Each implantable NGC consisted of two parts: an inner pDNM gel-coated or non-coated electrospun PLLA nanofibrous mesh and an outer P(LLA-TMC) protection tube. PLLA nanofibers were first obtained by electrospinning. These nanofibers were either randomly oriented (PLLA-random, Figure 1A) or longitudinally aligned (PLLA-aligned, Figure 1B) in 2D meshes. PLLA-aligned were then coated with pDNM gel at two different pDNM gel concentrations, which resulted in two distinct morphologies on the upper surface. When PLLA-aligned were decorated with 0.25% (w/v) pDNM gel (PLLA-aligned/0.25% pDNM gel, Figure 1C), large PLLA nanofibers (diameter = ~650 nm) were easily visualized by scanning electron microscopy (SEM) and were associated with much smaller nanofibrous structures (30-50 nm), which is attributable to collagen fiber assembly in the pDNM gel. When 1% (w/v) pDNM gel was applied (PLLA-aligned/1% pDNM gel, Figure 1D), the dECM hydrogel fully covered the surface of the electrospun mesh and the nanofibrous topography was hardly identifiable by SEM, which is consistent with our previous findings 32. The PLLA nanofibrous meshes, with or without pDNM gel coatings, were carefully wrapped around a stainless steel rod (diameter = 2 mm), then used as the collector for fabrication of electrospun P(LLA-TMC)70/30 protection tubes (illustrated in Figure 1E). The protection tubes possessed much denser fibers, which provides mechanical support, scaffold protection, and suturing capability (Figure 1F-G; microscopic morphology characterization, Figure S1; mechanical characterization, Figure S2).

### Nerve guidance of DRG explants in vitro

**Figure 2:**
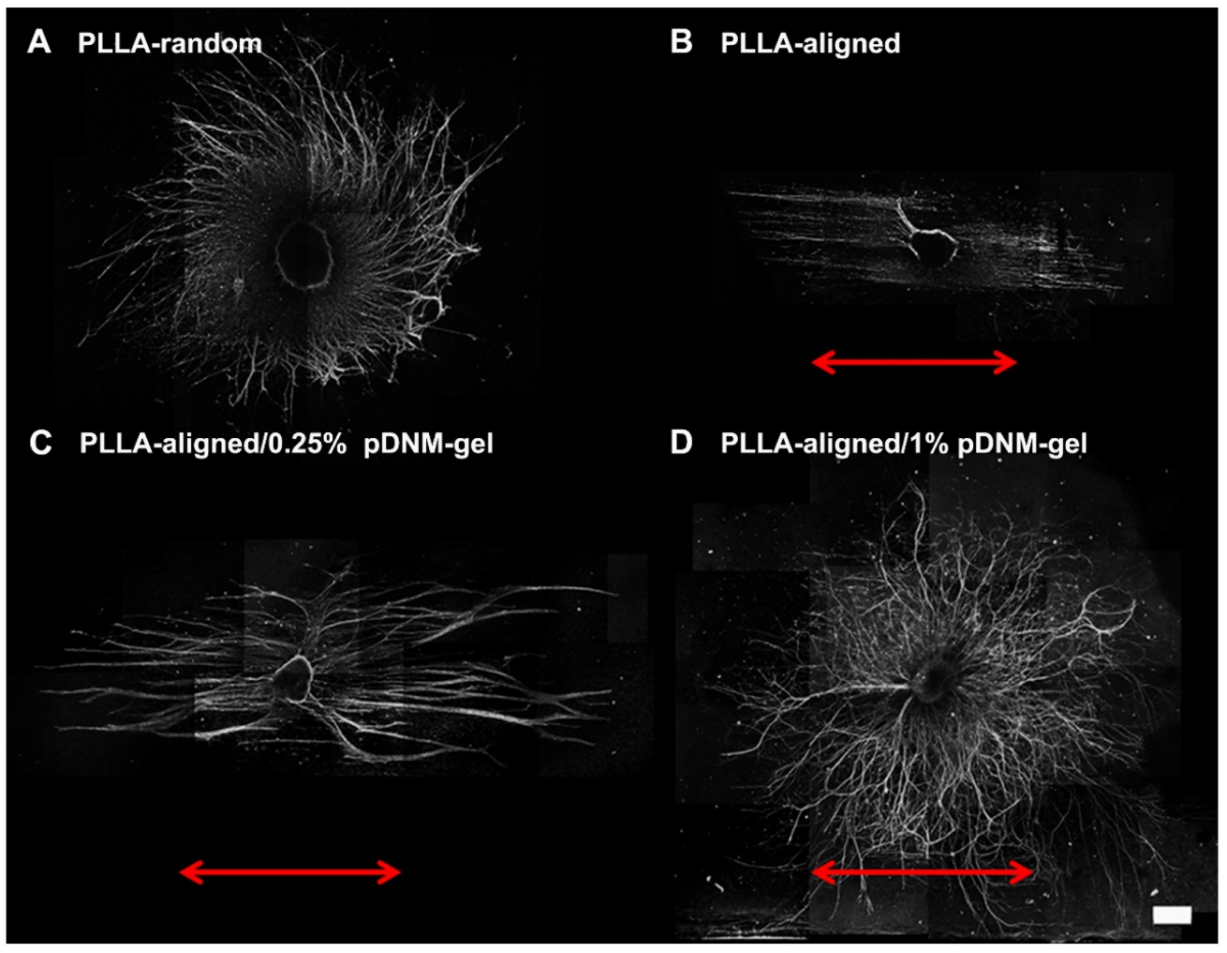
** Immunofluorescence images showing neurite sprouting and extension from DRG explants.** DRG explants were cultured on **(A)** PLLA-random, **(B)** PLLA-aligned, **(C)** PLLA-aligned/0.25% pDNM gel, or **(D)** PLLA-aligned/1% pDNM gel scaffolds. The red arrows indicate the direction of aligned nanofibers. The DRG explants were cultured for 7 days and immunostained for NF200. Scale bar = 500 μm.

The nerve guidance effect of each nanofibrous scaffold was examined by DRG culture. Regenerating axons (NF200+) grew radially from seeded DRG explants when cultured on PLLA-random and PLLA-aligned/1% pDNM gel scaffolds, indicating an absence of topological guidance (Figure 2A, D). In comparison, neurite outgrowth was highly oriented along the electrospun nanofibers in both the PLLA-aligned and PLLA-aligned/0.25% pDNM gel groups, with integration of pDNM gel leading to much longer and thicker neurites (Figure 2B-C). Similar trends were observed for Schwann cells (S100+) migration on the fabricated scaffolds, with a larger number of Schwann cells migrating further on the pDNM gel-coated scaffolds than on the PLLA alone meshes (Figure S3). These *in vitro* results are consistent with our previous findings 23, but their outcomes on nerve regeneration and functional recovery still remain to be evaluated *in vivo*.

### MRI monitoring of PNI repair in vivo

**Figure 3:**
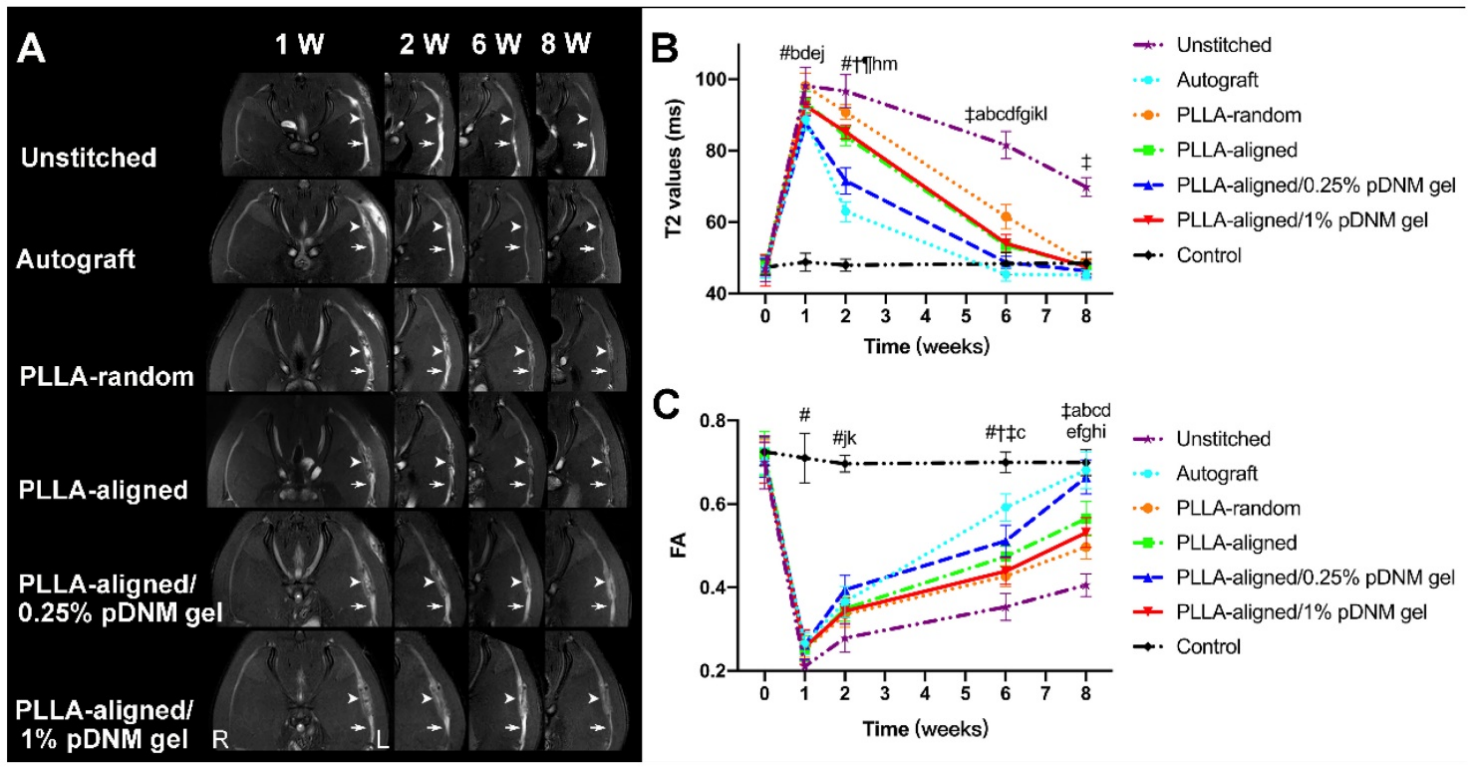
** Sequential FS-T2WI of injured and contralateral uninjured sciatic nerves in rats. (A)** MRI images showing the injured nerves on the left side of the unstitched, autografted, PLLA-random, PLLA-aligned, PLLA-aligned/0.25% pDNM gel, and PLLA-aligned/1% pDNM gel groups, and their corresponding uninjured nerves (control group) on the contralateral side. The injured nerves in all groups showed obviously enlarged and hyperintense signals in the proximal and distal portions, which gradually settled over time. The implanted NGCs (pointed out by arrowheads) were clearly delineated and hyperintense. R: right, L: left, W: week. **(B)** T2 values in the distal stumps of the injured nerves of all groups compared with their controls up to 8 weeks post-surgery. Statistically significant differences are listed in Table S2. **(C)** FA values in the distal stumps of the injured nerves of all groups compared with their controls up to 8 weeks post-surgery. Statistically significant differences are listed in Table S3.

After surgery, the transected sciatic nerves in the unstitched group, the grafted sciatic nerves of the groups implanted with NGCs, and the autografted nerves were clearly visualized by FS-T2WI (Figure 3A). The uninjured nerves on the contralateral side served as the control group. The distal stumps of the injured nerves exhibited obviously enlarged and hyperintense T2 signals in all groups at the first week after surgery, which gradually decreased. The T2 signals of the autografted group and the PLLA-aligned/0.25% pDNM gel group returned to normal (similar to the uninjured nerves) at 6 weeks post-operation, while the groups implanted with the other NGCs took 8 weeks to return to normal. The T2 signals of the injured nerves in the unstitched group remained discontinuous two months after surgery.

The time dependence of the T2 values in the distal stumps of the injured nerves varied across the unstitched, autografted, PLLA-random, PLLA-aligned, PLLA-aligned/0.25% pDNM gel, and PLLA-aligned/0.25% pDNM gel groups (Figure 3B). Consistently, the T2 values of all groups implanted with NGCs and the autografted group increased rapidly and reached similar peak values after one week. Afterwards, they decreased gradually but at different rates (slopes are given in Figure 3B). The T2 values of the autografted group and the PLLA-aligned/0.25% pDNM gel group reached their lowest levels in only 6 weeks, while the T2 values of the other injured groups continued to decrease for another two weeks. The T2 values of the autografted group were the lowest among all groups at 2 to 6 weeks post-operation. The T2 values of the PLLA-aligned group were lower than those of the PLLA-random group at 2 to 8 weeks. Additionally, the pDNM gel coating concentration influenced the T2 curves. The T2 values of the PLLA-aligned/0.25% pDNM gel group were significantly lower than those of the PLLA-aligned group at the second week post-surgery. However, the PLLA-aligned/1% pDNM gel group showed higher T2 values than the PLLA-aligned group within the same period, most likely due to the loss of topological guidance provided by the electrospun nanofibers. The T2 values of the unstitched group were the highest of all groups at 2 to 8 weeks and remained much higher than the normal level afterwards.

The time dependence of the FA values followed opposite trends of the T2 values for all groups (Figure 3C). At 2 to 8 weeks after surgery, the FA values continued to increase, implying axonal regeneration within the injured nerves. Unlike the T2 values, the FA values of the injured nerves gradually approached but remained lower than those of the contralateral uninjured nerves at 8 weeks post-surgery. All the groups treated with aligned nanofibers, with or without pDNM gel coatings, exhibited larger FA values than those of the PLLA-random group at the first week after surgery. Among the groups treated with aligned nanofibers, the PLLA-aligned/0.25% pDNM gel group maintained the largest FA values at 2, 6, and 8 weeks post-operation, while the FA values of the PLLA-aligned/1% pDNM gel group were the lowest.

**Figure 4:**
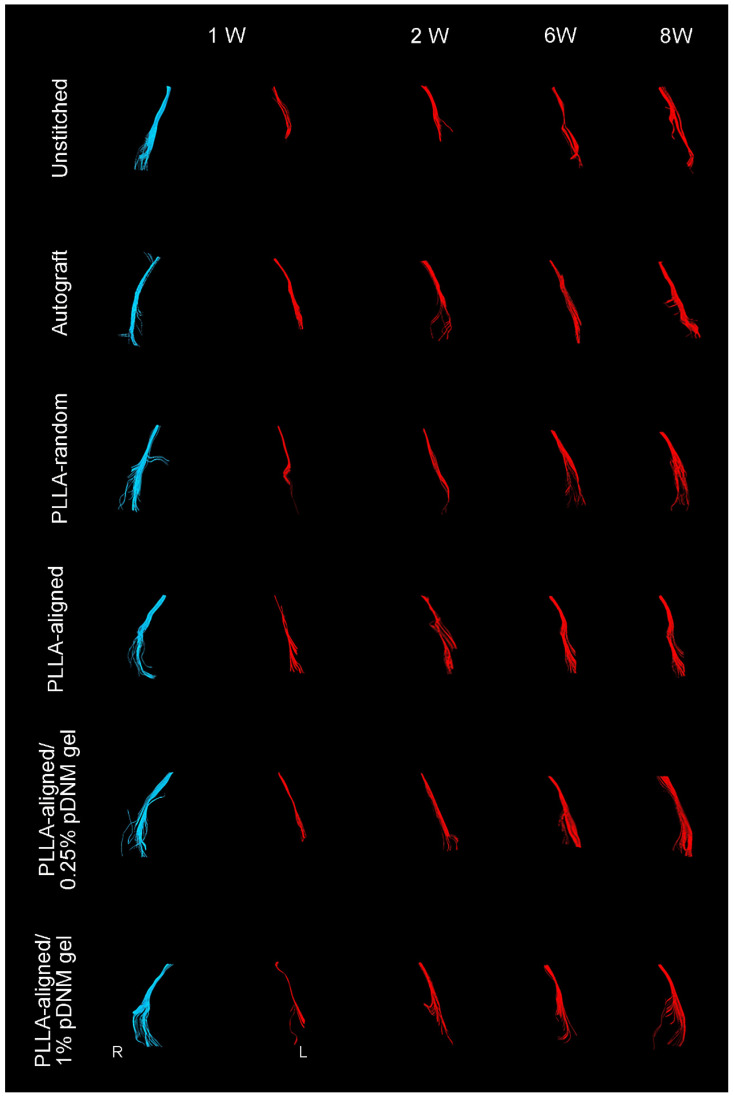
**DTT of injured sciatic nerves (red) after surgery compared with uninjured nerves on the contralateral side (blue).** Representative images showing the injured nerves on the left side of the unstitched, autografted, PLLA-random, PLLA-aligned, PLLA-aligned/0.25% pDNM gel, and PLLA-aligned/1% pDNM gel groups, and their corresponding uninjured nerves (control group) on the contralateral side. R: right, L: left, W: week.

Compared with their contralateral uninjured nerves, the diameter of nerve tracts and the number of projected injured nerve fibers decreased 2 weeks after surgery, as characterized by DTT analysis (Figure 4). Thereafter, the morphology of all grafted nerves gradually recovered. The defected nerves almost returned to normal in the autografted group by 8 weeks post-operation. The PLLA-aligned/0.25% pDNM gel group showed more nerve fibers and larger nerve trajectories than the groups implanted with other NGCs at 2 to 8 weeks after surgery. The unstitched nerves remained discrete after surgery.

### Histological characterization of nerve regeneration

**Figure 5:**
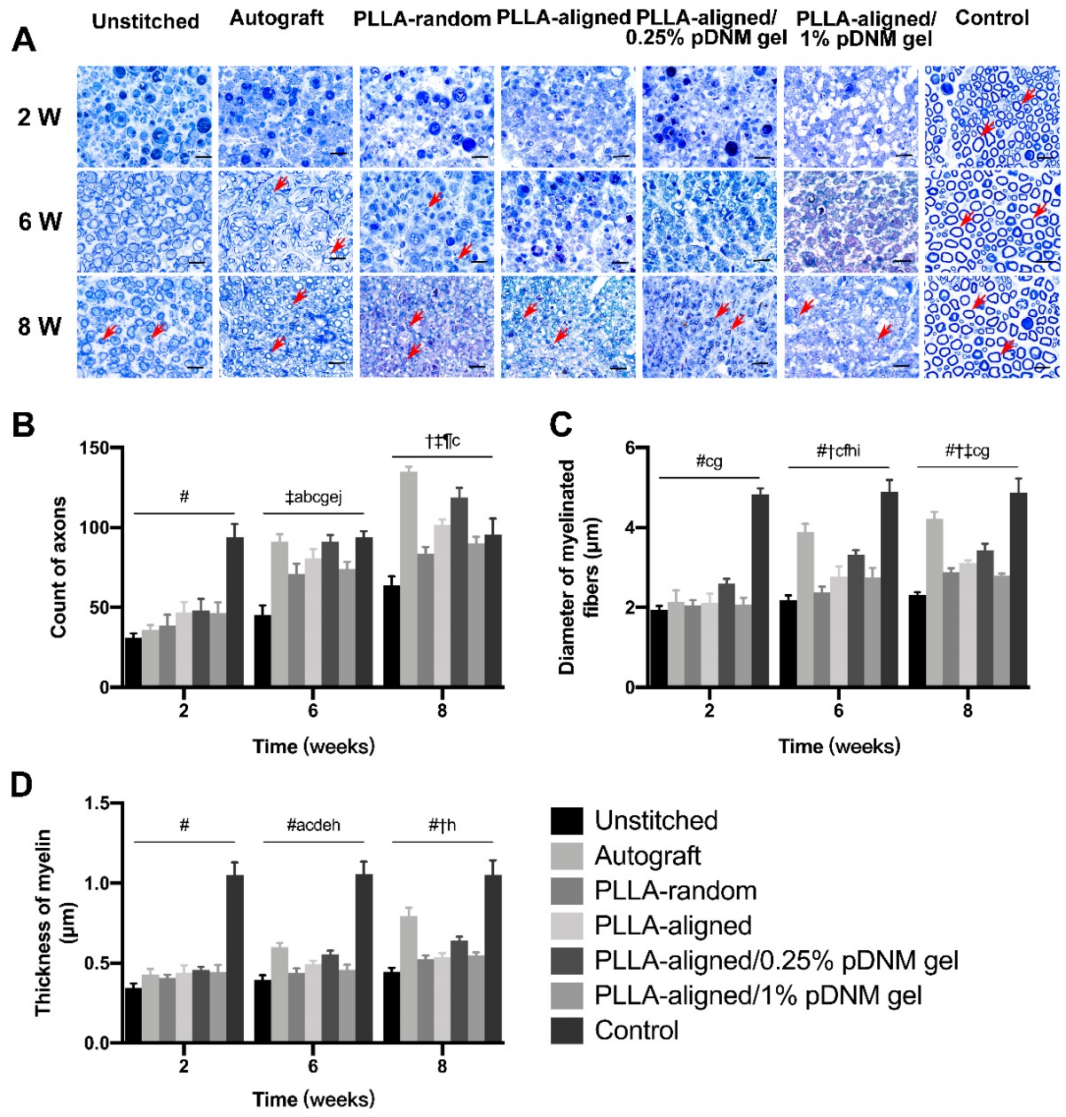
** Toluidine blue staining of the distal stumps of injured and contralateral uninjured nerves. (A)** Representative cross-sectional micrographs exhibiting axonal degeneration and myelin disintegration at 2 weeks post-surgery. The red arrowheads point out the myelinated axons. Pronounced axonal regeneration and remyelination were observed at 6 and 8 weeks post-operation, especially in the autografted group and the PLLA-aligned/0.25% pDNM gel group. W: week. Scale bars = 20 μm. Number of axons **(B)**, myelin thickness **(C)**, and diameter of myelinated nerve fibers **(D)** in the distal stumps of the injured nerves of all groups compared with their contralateral uninjured nerves (control group) at 2, 6, and 8 weeks post-surgery. Statistically significant differences are listed in Table S4.

Toluidine blue staining of the distal stumps of the injured nerves revealed massive axonal degeneration and myelin destruction 2 weeks post-operation. After treatment, significant progress in myelin debris clearance, axonal regeneration, and remyelination was identified in all groups at 6 and 8 weeks after surgery (Figure 5A). This was also evident within the injured portion (center region of the nerve grafts) at 8 weeks post-operation (Figure S4A). Statistically, the autografted group showed the largest number of axons (Figure 5B) as well as the most significant myelination effects at 6 and 8 weeks, including myelin thickness (Figure 5C) and myelinated nerve fiber diameter (Figure 5D), at the distal stumps among all injured groups, followed by the PLLA-aligned/0.25% pDNM gel group. Additionally, the PLLA-aligned/0.25% pDNM gel group showed the greatest number of axons and most significant myelination effects at the injured portion of the lesion sites among all the groups implanted with NGCs at 8 weeks (Figure S4B). Without pDNM gel, the topological guidance provided by the longitudinally aligned nanofibers in the PLLA-aligned group highly promoted nerve regeneration (axons count) and nerve fiber myelination (thickness of myelin and diameter of myelinated fibers), compared to the PLLA-random group. Though incorporation of pDNM gel facilitates Schwann cells migration, the high gel concentration in the PLLA-aligned/1% pDNM gel group attenuated the topological guidance effects provided by the electrospun nanofibers, which resulted in almost equivalent or even fewer regenerative axons and thinner myelin compared to the PLLA-aligned group (without pDNM gel) at 6 and 8 weeks post-surgery.

**Figure 6:**
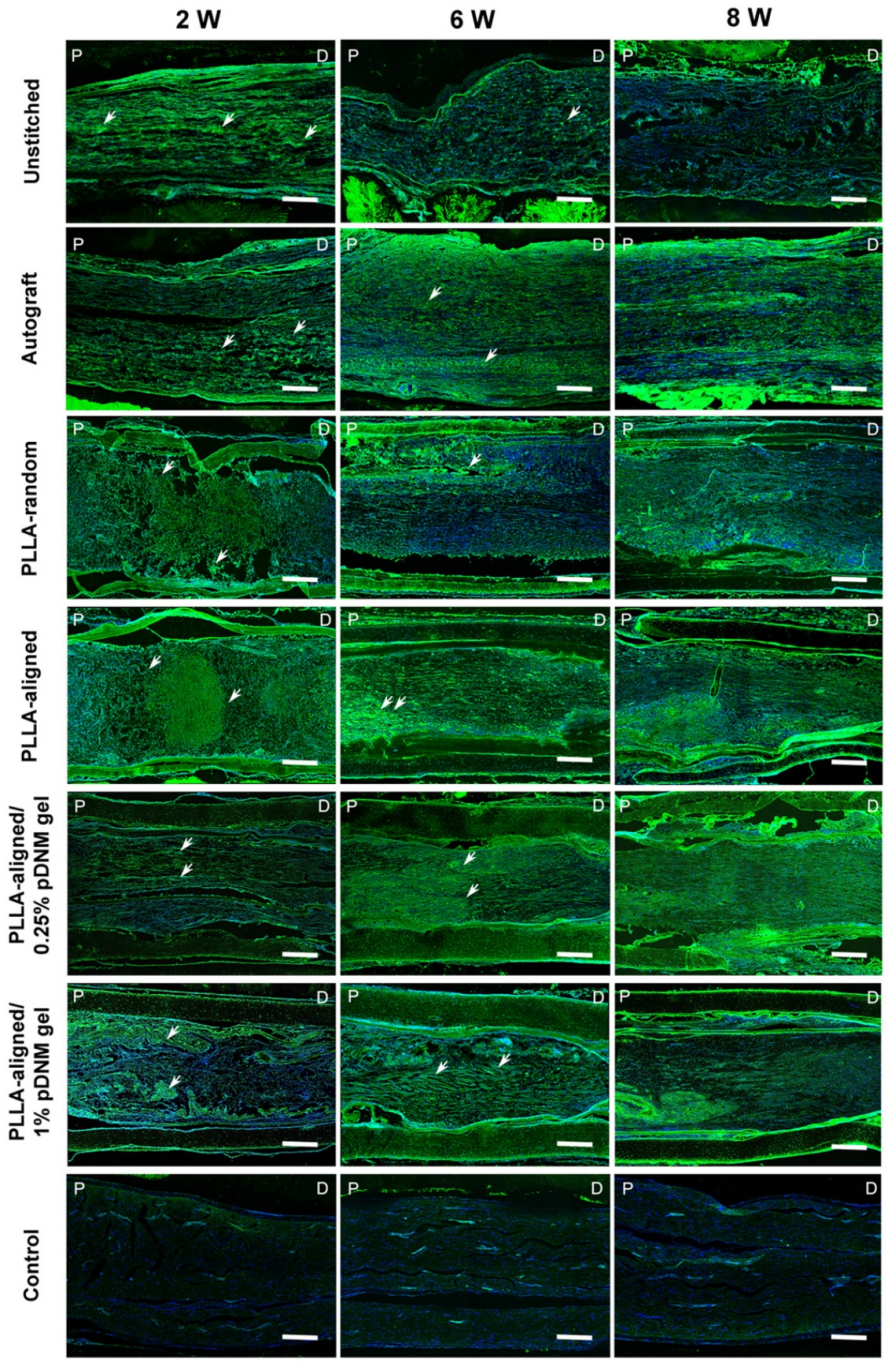
** SPRR1A staining showing the continuity of regenerating nerve fibers.** Representative images of regrowing axons from all groups, immunofluorescence stained with SPRR1A at 2, 6, and 8 weeks post-surgery. The white arrowheads point out the regrowing axons. P: proximal, D: distal, W: week. Scale bars = 300 μm.

The continuity of injured and regenerating nerve fibers was assessed by immunofluorescence staining using biomarker SPRR1A for regrowing axons (Figure 6). At the early stage after treatment (2 weeks post-operation), the nerve fibers were highly disordered at the injured sites of the unstitched, PLLA-random, PLLA-aligned, and PLLA-aligned/1% pDNM gel groups, but longitudinally aligned in the autografted and PLLA-aligned/0.25% pDNM gel NGCs groups. After 6 and 8 weeks of nerve repair, nerve fiber continuity was significantly improved in almost all the grafted groups. The autografted group showed the most promising recovery in nerve fiber continuity, while the unstitched group exhibited the most disconnected nerve fibers. The alignment of nerve fibers in the PLLA-aligned group was more obvious compared to that of the PLLA-random and PLLA-aligned/1% pDNM gel groups. In comparison, in the PLLA-aligned/0.25% pDNM gel group, nerve fiber regrowth was closely packed and highly ordered, revealing the best performance in nerve regeneration. SPRR1A+ signal was minimal in the uninjured control group, since nerve fiber continuity was complete with very few growing axons.

### Motor functional recovery

**Figure 7:**
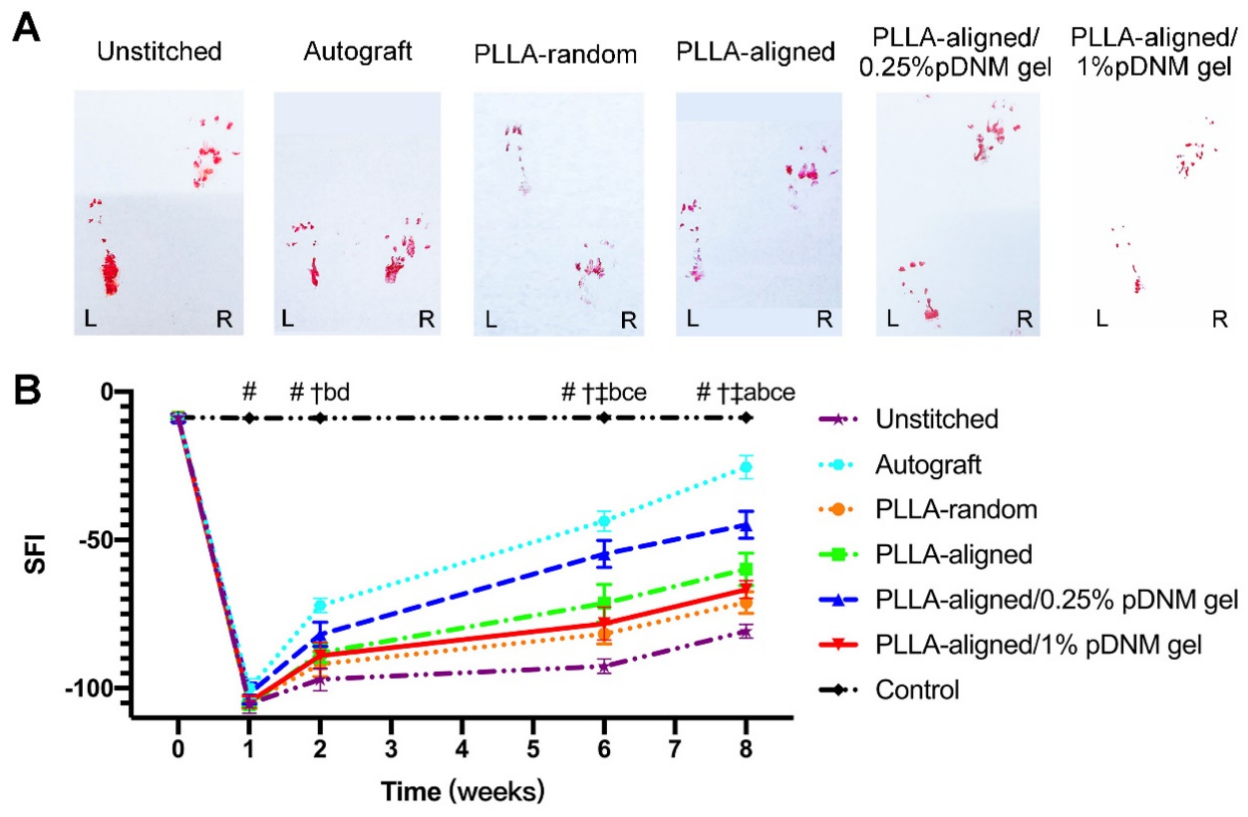
** Walking track analysis of the sciatic nerve-injured rats. (A)** Hind paw footprints of all injured groups at 8 weeks post-surgery. (B) Sciatic nerve function index (SFI) of the injured side compared with the contralateral uninjured side post-surgery. Statistically significant differences are listed in Table S5.

Finally, the motor function recovery of the injured rats was evaluated by walking track analysis and SFI. The hind paw footprints of the injured rats were recorded (Figure 7A). The injured animals displayed a sudden drop in SFI within the first week after surgery, which gradually recovered (Figure 7B). The SFI values kept increasing for all injured groups, with the autografted group possessing the highest SFI value at 2 to 8 weeks after surgery, followed by the PLLA-aligned/0.25% pDNM gel group. The SFI values of the PLLA-random group were -81.7 ± 3.4 and -71.1 ± 3.6 at 6 and 8 weeks, respectively (Table S5), while those of the PLLA-aligned/1% pDNM gel group were -78.2 ± 5.5 and -66.8 ± 3.0. The SFI values of these two groups were smaller than those of the PLLA-aligned (-71.4 ± 6.3 and -59.9 ± 5.5) and PLLA-aligned/0.25% pDNM gel groups (-54.7 ± 4.6 and -44.9 ± 4.5). These results indicate that the grafted NGCs without topological fiber guidance (PLLA-random and PLLA-aligned/1% pDNM gel groups) provided much slower functional restoration at 6 and 8 weeks post-surgery.

## Discussion

NGCs with aligned micro- or nano-architectures possess remarkable assets for promoting peripheral nerve regeneration 46-48. Surface topographical patterns are critical to neurite outgrowth, which not only triggers neurite formation but also serves as directional guidance for targeted axonal extension. NGCs made of electrospun nanofibers promote directed axonal extension owing to their anisotropic alignment, which serves as the topological guidance for nerve fiber growth as well as the migration of Schwan cells along the direction of axonal extension 23, 31, 32. In this study, longitudinally aligned electrospun nanofibers effectively provided guidance for neural cells. Histological analysis showed that the aligned nanofibrous structure in the PLLA-aligned group was a potent stimulator for axonal regeneration compared to the randomly oriented nanofibers in the PLLA-random group. Furthermore, the myelin thickness of the PLLA-aligned group was 0.494 ± 0.021 μm and 0.536 ± 0.028 μm at 6 and 8 weeks post-surgery, respectively, whereas the myelin thickness of the PLLA-random group was 0.440 ± 0.027 μm and 0.524 ± 0.028 μm. Nerve fiber remyelination was facilitated by the aligned nanofibers rather than the randomly oriented fibers (*p*<0.002)*.* As a result, implantation of NGCs composed of PLLA-aligned scaffolds performed more significant functional recovery than those composed of PLLA-random.

Synthetic materials, such as PLLA 27, poly(ε-caprolactone) 24, and other copolymers 31, have been applied for fabrication of tissue-engineered nerve scaffolds due to their mechanical stability and reproducibility 6. However, topological guidance provided by electrospun nanofibers alone cannot fully recapitulate or mimic the complex microenvironment of nerve cells due to their lack of structural ECM components and neurotrophic factors. Incorporation of ECM proteins (e.g., collagen, laminin, fibronectin) or growth factors (e.g., nerve growth factor) into electrospun nanofibers has been frequently investigated in recent years 27, 49-51. NGCs fabricated using these composite materials often have limited capability for nerve functional restoration, either because of their inferior bioactivity or rapid release of the bioactive compounds from the nanofibers. It is widely realized that dECM biomaterials obtained directly from tissues or organs may reconstruct a well-defined physical and chemical microenvironment for cell survival and development, and determine morphogenesis, homeostasis, and stem cell differentiation in tissue repair 11. A recent study verified that porcine peripheral nerve-derived dECM scaffolds can effectively suppress immunoreactions after implantation, which was mostly attributed to the elimination of cellular components 52. After processing into a hydrogel, the resulting pDNM gel preserved most of the functional ECM components, including glycosaminoglycans, collagens, laminin, and fibronectin, which actively promote neurite outgrowth 22, 37. Furthermore, pDNM gel was shown to facilitate nerve fiber remyelination in vitro 37. However, pDNM gel alone could neither provide enough mechanical robustness to enable implantation, nor topological guidance for directed axonal extension. Therefore, the combination of electrospun PLLA nanofibers and pDNM gel are beneficial for nerve regeneration, which was proven *in vitro* as well as *in vivo* in this study. We found that NGCs composed of PLLA-aligned/0.25% pDNM gel exhibited superior performance in repairing peripheral nerve defects in terms of axonal regeneration, extent of myelination, and functional recovery compared to either PLLA-aligned/1% pDNM gel conduits or NGCs composed of aligned PLLA nanofibers alone (without pDNM gel coating). These results reveal that the fully shielded nanofibrous structures in the PLLA-aligned/1% pDNM gel scaffold concealed the topological guidance, which led to attenuated nerve regeneration despite the increased pDNM gel concentration.

Multiparametric MRI was employed to assess the extent of axonal regeneration in situ. T2 and FA values can serve as sensitive indicators for detecting peripheral nerve injury, as well as appropriate tools for noninvasive monitoring of nerve regeneration. T2 hyperintensity of distal degenerating nerves is the result of increased water content, myelin turnover, vascular permeability, inflammatory mediators, and axonal and myelin breakdown products 53, 54. Meanwhile, FA values, as the main quantitative parameters of DTI, are more strongly correlated with the density and diameter of axons, rather than the density and thickness of myelin 43. Quantitative T2 values and DTI metrics can be further compared with histological results and behavioral assessments for a fuller picture of axonal regeneration. In this study, the T2 and FA values of injured nerves grafted with NGCs composed of PLLA-aligned/0.25% pDNM gel indicated accelerated nerve tissue recovery compared with those of the PLLA-aligned and PLLA-aligned/1% pDNM gel groups. As histological assessments showed that more neurites, thicker axons, and thicker myelin were evident within regenerating nerve fibers of the PLLA-aligned/0.25% pDNM gel group compared to the other grafted groups, the rapid recoveries of T2 and FA values were most likely due to promoted axonal regeneration. Notedly, the nerve defects did not fully recover in all groups by 8 weeks post-surgery based on the histological analysis. However, the T2 values returned to normal, or even lower than those of the control group (i.e., uninjured nerve on the contralateral side), whereas the FA values were still much lower than normal in all grafted groups at 1 to 8 weeks after surgery. This result implies that the FA values were more sensitive than the T2 values for detecting nerve regeneration.

FA measures the degree of anisotropic diffusion, which reflects the degree of cell alignment within the nerve fiber tracts. The FA value varies from 0 to 1, where 0 represents isotropic diffusion and 1 represents complete anisotropy 55. In previously reported nerve crush injury models, increased FA values were observed within 3 weeks after injury in rats, while it took 4 weeks for the FA value to begin increasing after sciatic nerve injury in rabbits 56, 57. These time points indicate the termination of Wallerian degeneration and complete clearance of myelin and axon debris 43. In this study, the FA values began to increase only after the first week post-surgery for all groups implanted with NGCs, which is when the damaged nerves entered the axonal regeneration phase. However, the FA values of the PLLA-aligned/0.25% pDNM gel group increased much faster than those of the other groups implanted with NGCs, especially at 6 to 8 weeks post-surgery. Though the final FA values did not reach the levels of the uninjured contralateral nerves or the autografted group, the PLLA-aligned/0.25% pDNM gel group was considerably close to complete recovery at 8 weeks. These FA value changes were consistent with the behavioral and histological results. Taken together, these findings suggest that MRI assessment may serve as a useful tool for evaluating PNI repair.

## Conclusions

NGCs incorporating aligned electrospun nanofibers providing topological guidance combined with pDNM gel inducing biological cues were implanted into a defected sciatic nerve injury rat model. Multiparametric MRI including T2-mapping and DTI was used to dynamically monitor the extent of nerve regeneration. The aligned nanofibers exhibited excellent performance in triggering directed axonal extension compared with randomly oriented nanofibers. Surface decoration with pDNM gel at a relatively low concentration further promoted nerve regeneration, including the number of axons, continuity of the nerve fibers, remyelination, and functional recovery. This biomaterial scaffold combined with MRI assessment is a promising approach for neural tissue engineering and the treatment and diagnosis of PNI.

## Supplementary Material

Supplementary materials and methods, figures and tables.

## Abbreviations

PNI: peripheral nerve injury
NGC: nerve guidance conduit
pDNM: porcine decellularized nerve matrix
*L*-lactic acid): PLLA, Poly
MRI: magnetic resonance imaging
FA: fractional anisotropy
SFI: sciatic nerve function index
dECM: decellularized extracellular matrix
DRG: dorsal root ganglion
SC: Schwann cell
DTI: diffusion tensor imaging
TFE: 2,2,2-trifluoroethanol
*L*-lactic acid-co-trimethylene carbonate): P(LLA-TMC
T2WI: T2-weighted imaging
TR: repetition time
TE: echo time
FOV: field of view
NSA: number of signals averaged
EPI: echoplanar imaging
FA: fractional anisotropy
ROI: region of interest
DTT: diffusion tensor tractography
SPRR1A: small proline-rich protein 1A
NPL: normal print length
NTS: normal toe spread
NIT: normal intermediary toe spread
EPL: experimental print length
ETS: experimental toe spread
EIT: experimental intermediary toe spread
