# Supplementary material for: Nanofibrous nerve guidance conduits decorated with decellularized matrix hydrogel facilitate peripheral nerve injury repair
Source: Theranostics. 2021 Jan 1;11(6):2917–31. doi: 10.7150/thno.50825 (PMC7806490; doi:10.7150/thno.50825)
Supplement: Supplementary file 1 — Supplementary materials and methods, figures and tables. [file thnov11p2917s1.pdf]

Supplementary Materials and Methods

Mechanical characterization of the NGC protection tube

The mechanical property of NGC was tested by a dynamic mechanical analysis apparatus (1dB-Metravib, France). The prepared P(LLA-TMC) tubes (with length, *L*) was placed between and slightly in contact with the upper and lower compression clamps. The initial distance between the clamps was D_0. The force between the clamps was gradually increased at rate ~0.05 N/s, and the maximum stroke was set to 1.5 mm. The real-time force *F* and displacement *D* at each point were recorded. A measurement curve was drawn using the force per unit length (F/L) as the ordinate, and the percentage of deformation (D/D_0) as the abscissa. The force per unit length with 10% deformation was measured and analyzed (n = 4).

Supplementary Figures

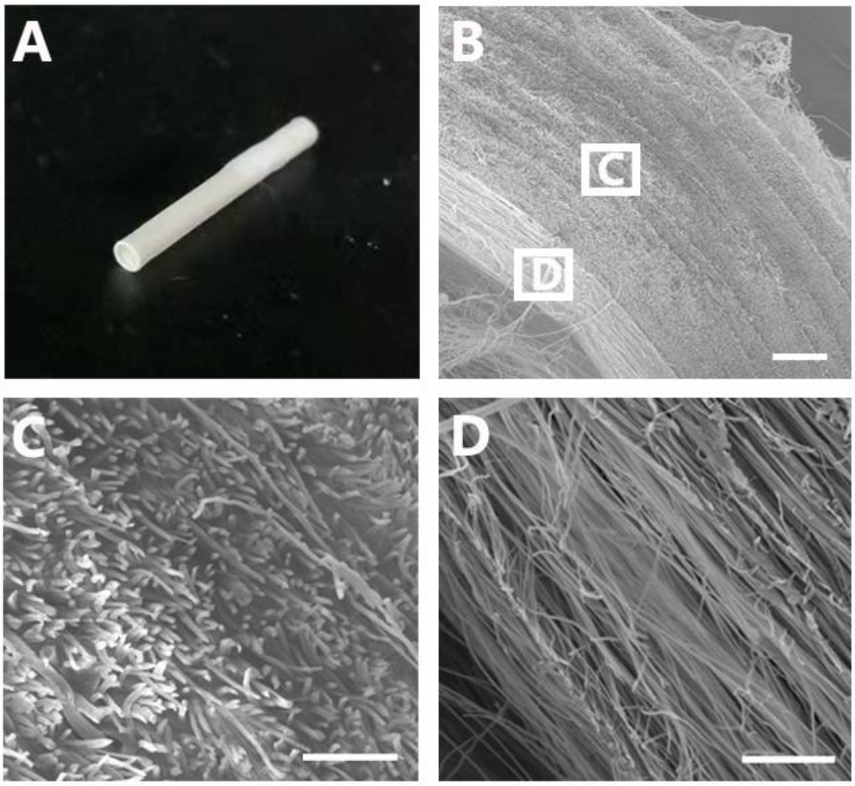

Figure S1. P(LLA-TMC) protection tube for NGC fabrication. (A) A representative photograph of NGC for implantation after PNI. **(B)** SEM image showing ~1/4 part of cross-sectional area in a typical NGC. Scale bar = 100 µm. **(C)** Representative cross-section of the protection tube, and (D) representative longitudinal view of PLLA-aligned scaffold (without pDNM gel coating). Scale bars = 20 µm.

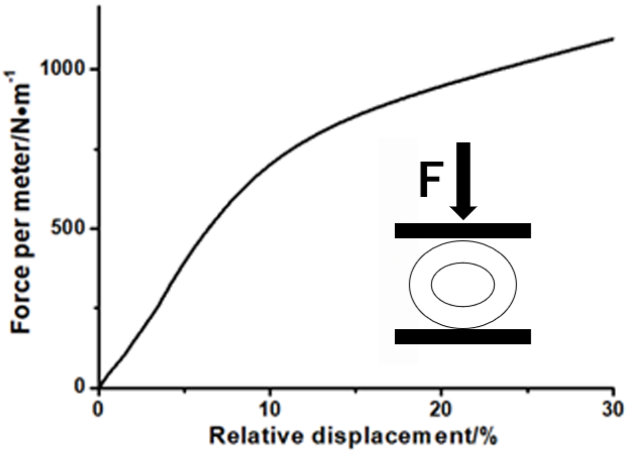

Figure S2. Compression test on an electrospun P(LLA-TMC) protection tube.

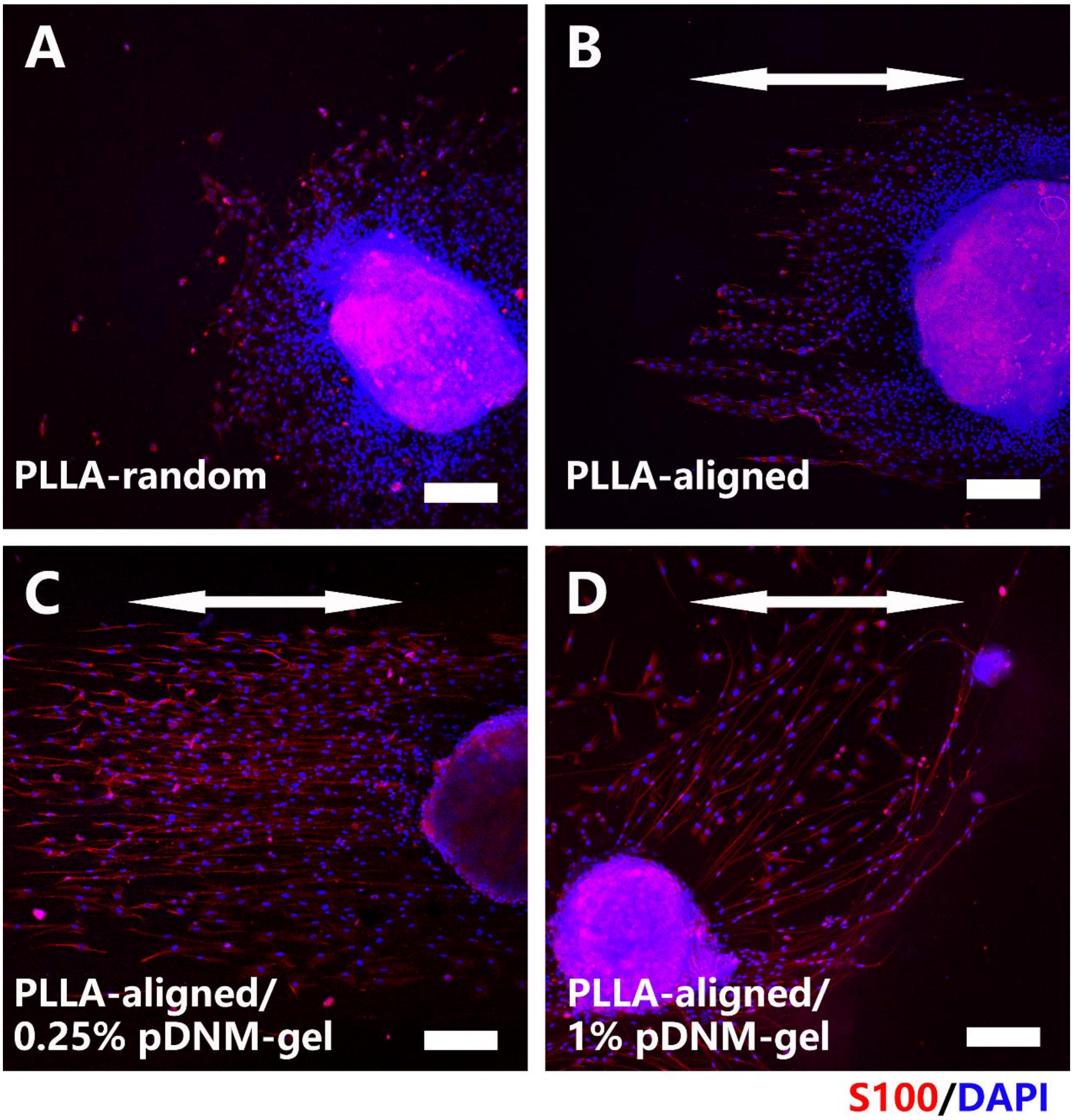

Figure S3. Immunofluorescence micrographs showing Schwann cells migration from DRG explants seeded on (A) PLLA-random, **(B)** PLLA-aligned, **(C)** PLLA-aligned/0.25% pDNM gel, and **(D)** PLLA-aligned/1% pDNM gel scaffolds, respectively. The white arrows point out the direction of aligned nanofibers. The DRG explants were cultured for 7 days and immunostained by S100 and DAPI. Scale bars = 200 μm.

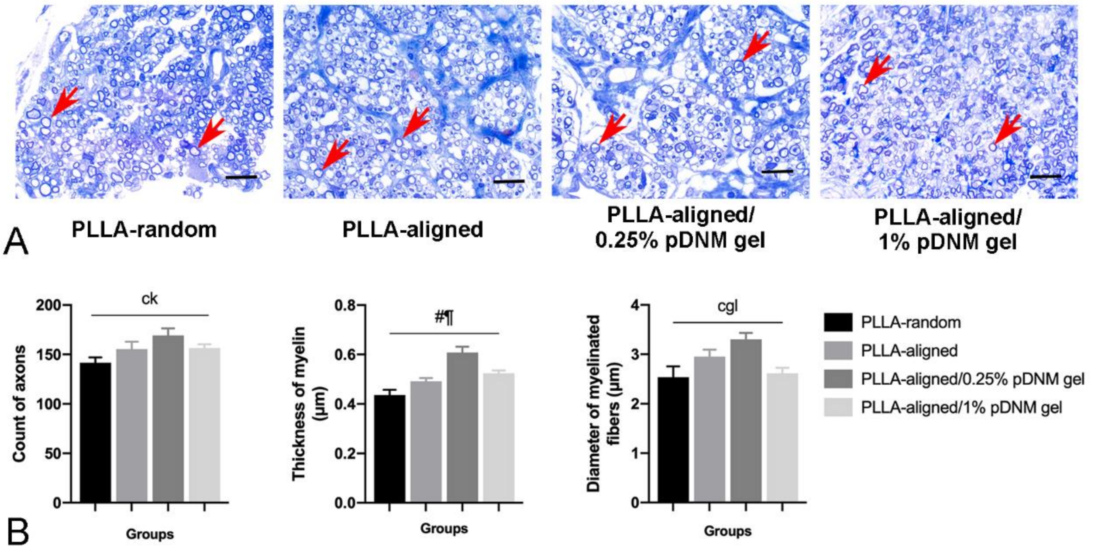

Figure S4. **Toluidine blue staining on the injured portion (center region of the nerve grafts) in the NGC implanted groups. (A)** Representative cross-sectional micrographs show axonal degeneration/regeneration and myelin disintegration/formation 8 weeks post-surgery. The red arrowheads point out the myelinated axons. **(B)** Number of axons, thickness of myelin, and diameter of myelinated nerve fibers in injured portion of the graft nerves of all NGC implanted groups. Statistically significant differences are listed in Table S4.

Supplementary Tables

Table S1. Diameters of nanofibers in the nerve scaffold of each group

Scaffold	PLLA nanofibers/nm	pDNM gel nanofibers/nm
PLLA-random	820±90	-
PLLA-aligned	650±90	-
PLLA-aligned/0.25% pDNM gel	650±90	40±10
PLLA-aligned/1% pDNM gel	-	-

Table S2. T2 values in Unstitched, Autograft, PLLA-random, PLLA-aligned, PLLA-aligned/0.25% pDNM gel, PLLA-aligned/1% pDNM gel, and Control groups

Time points (weeks)	Unstitched (mean ± SD)	Autograft (mean ± SD)	PLLA-random (mean ± SD)	PLLA-aligned (mean ± SD)	PLLA-aligned/0.25% pDNM gel (mean ± SD)	PLL-aligned/1% pDNM gel (mean ± SD)	Control (mean ± SD)	$p < 0.002$
0 (Pre)	46.91 ± 3.68	47.15 ± 2.87	47.67 ± 3.26	47.93 ± 2.23	47.33 ± 2.45	46.17 ± 2.92	47.35 ± 1.98	-
1	98.18 ± 5.17	88.70 ± 3.06	98.08 ± 3.60	93.62 ± 2.12	88.38 ±1.89	93.15 ± 3.28	48.73 ± 2.48	#bdej
2	96.65 ± 4.59	62.92 ± 2.77	90.77 ± 2.06	84.03 ± 2.72	71.50 ± 3.65	85.18 ± 1.87	47.93 ± 1.68	#†¶hm
6	81.53 ± 3.82	45.35 ± 1.90	61.45 ± 3.41	53.40 ± 1.71	46.82 ± 2.09	61.97 ± 4.84	48.33 ± 3.08	‡abcdfgikl
8	69.78 ±2.66	45.17 ±1.31	48.30 ±1.53	47.72 ± 1.08	46.33 ±1.86	47.53 ± 2.02	48.43 ± 3.10	‡

SD: standard deviation; $p < 0.002$

Statistically significant difference between
#: Group control vs. all the other groups
†: Group autograft vs. all the other groups
‡: Group unstitched vs. all the other groups
¶: Group PLLA-aligned/0.25% pDNM gel vs. all the other groups
[a]Group PLLA-random vs. PLLA-aligned;
[b]Group PLLA-random vs. autograft;
[c]Group PLLA-random vs. control;
[d]Group PLLA-random vs. PLLA-aligned/0.25% pDNM gel
[e]Group autograft vs. unstitched
[f]Group autograft vs. PLLA-aligned
[g]Group autograft vs. PLLA-aligned/1% pDNM gel
[h]Group PLLA-aligned vs. unstitched
[i]Group PLLA-aligned vs. PLLA-aligned/1% pDNM gel
[j]Group PLLA-aligned/0.25% pDNM gel vs. unstitched
[k]Group PLLA-aligned/0.25% pDNM gel vs. PLLA-aligned/1% pDNM gel
[l]Group PLLA-aligned/1% pDNM gel vs. control
[m]Group PLLA-aligned/1% pDNM gel vs. unstitched

Table S3. FA values in Unstitched, Autograft, PLLA-random, PLLA-aligned, PLLA-aligned/0.25% pDNM gel, PLLA-aligned/1% pDNM gel, and Control groups

Time points (weeks)	Unstitched (mean ± SD)	Autograft (mean ± SD)	PLLA-random (mean ± SD)	PLLA-aligned (mean ± SD)	PLLA-aligned/0.25% pDNM gel (mean ± SD)	PLLA-aligned/1% pDNM gel (mean ± SD)	Control (mean ± SD)	$p < 0.002$
0 (Pre)	0.700 ± 0.063	0.703 ± 0.032	0.712 ± 0.043	0.721 ± 0.053	0.706 ± 0.042	0.698 ± 0.048	0.725 ± 0.034	-
1	0.210 ± 0.016	0.266 ± 0.026	0.250 ± 0.017	0.254 ± 0.031	0.263 ± 0.035	0.257 ± 0.041	0.710 ± 0.059	#
2	0.279 ± 0.034	0.365 ± 0.038	0.336 ± 0.031	0.349 ± 0.030	0.394 ± 0.034	0.343 ± 0.031	0.696 ± 0.020	#jk
6	0.353 ± 0.033	0.592 ± 0.032	0.427 ± 0.025	0.474 ± 0.024	0.519 ± 0.020	0.439 ± 0.031	0.700 ± 0.025	#†‡c
8	0.406 ± 0.027	0.682 ± 0.044	0.497 ± 0.029	0.565 ± 0.042	0.665 ± 0.040	0.531 ± 0.036	0.699 ± 0.031	‡abcdefghi

SD: standard deviation; $p < 0.002$

Statistically significant difference between
#: Group control vs. all the other groups
†: Group autograft vs. all the other groups
‡: Group unstitched vs. all the other groups
[a]Group PLLA-random vs. autograft;
[b]Group PLLA-random vs. control;
[c]Group PLLA-random vs. PLLA-aligned/0.25% pDNM gel
[d]Group autograft vs. PLLA-aligned
[e]Group autograft vs. PLLA-aligned/1% pDNM gel
[f]Group PLLA-aligned vs. control
[g]Group PLLA-aligned vs. PLLA-aligned/0.25% pDNM gel
[h]Group PLLA-aligned/0.25% pDNM gel vs. PLLA-aligned/1% pDNM gel
[i]Group PLLA-aligned/1% pDNM gel vs. control
[j]Group unstitched vs. autograft
[k]Group unstitched vs. PLLA-aligned/0.25% pDNM gel

Table S4. Histological characterizations in Unstitched, Autograft, PLLA-random, PLLA-aligned, PLLA-aligned/0.25% pDNM gel, PLLA-aligned/1% pDNM gel, and Control groups

**Histology of the injury nerves**	**Time points (weeks)**	**Unstitched (mean ± SD)**	**Autograft (mean ± SD)**	**PLLA-random (mean ± SD)**	**PLLA-aligned (mean ± SD)**	**PLLA-aligned/0.25% pDNM gel (mean ± SD)**	**PLLA-aligned/1% pDNM gel (mean ± SD)**	**Control (mean ± SD)**	***p***
**Count of axons**	2	31.0 ± 2.7	36.0 ± 2.7	38.8 ± 6.6	47.0 ± 6.4	48.2 ± 7.2	46.6 ± 6.6	93.8 ± 8.4	#
	6	45.4 ± 5.9	91.0 ± 4.3	71.0 ± 6.4	80.6 ± 5.9	91.0 ± 4.3	74.0 ± 4.6	93.8 ± 4.0	‡abcgej
	8	64.0 ± 5.4	135.0 ± 3.1	83.8 ± 4.1	101.8 ± 3.1	118.8 ± 6.0	90.2 ± 4.0	95.4 ± 10.2	†‡¶c
	8* (Injured portion)	-	-	141.6 ± 5.4	155.4 ± 7.4	169.2 ± 7.1	156.6 ± 3.7	-	ck
**Myelin thickness**	2	0.344 ± 0.028	0.430 ± 0.034	0.408 ± 0.019	0.438 ± 0.050	0.458 ± 0.019	0.442 ± 0.047	1.052±0.077	#
	6	0.396 ± 0.027	0.600 ± 0.025	0.440 ± 0.027	0.494 ± 0.021	0.556 ± 0.021	0.458 ± 0.031	1.056±0.078	#acdeh
	8	0.446 ± 0.024	0.794 ± 0.053	0.524 ± 0.028	0.536 ± 0.028	0.640 ± 0.026	0.548 ± 0.019	1.054±0.090	#†h
	8* (Injured portion)	-	-	0.436 ± 0.021	0.492 ± 0.013	0.608 ± 0.029	0.524 ± 0.011	-	#¶
**Myelinated fiber diameters**	2	1.932 ± 0.100	2.130 ± 0.298	2.054 ± 0.128	2.118 ± 0.222	2.598 ± 0.118	2.064 ± 0.177	4.822 ± 0.161	#cg
	6	2.182 ± 0.123	3.900 ± 0.198	2.368 ± 0.147	2.778 ± 0.240	3.316 ± 0.115	2.754 ± 0.235	4.898 ± 0.289	#†cfhi
	8	2.301 ± 0.075	4.226 ± 0.166	2.890 ± 0.092	3.110 ± 0.069	3.428 ± 0.167	2.800 ± 0.046	4.878 ± 0.347	#†‡cg
	8* (Injured portion)	-	-	2.538 ± 0.217	2.954 ± 0.139	3.304 ± 0.130	2.616 ± 0.112	-	cgl

SD: standard deviation; * $p < 0.008$; $p < 0.002$

Statistically significant difference between
#: Group control vs. all the other groups
†: Group autograft vs. all the other groups
‡: Group unstitched vs. all the other groups
¶: Group PLLA-aligned/0.25% pDNM gel vs. all the other groups
[a]Group PLLA-random vs. autograft;
[b]Group PLLA-random vs. control;
[c]Group PLLA-random vs. PLLA-aligned/0.25% pDNM gel
[d]Group autograft vs. unstitched
[e]Group autograft vs. PLLA-aligned/1% pDNM gel
[f]Group PLLA-aligned vs. unstitched

[g]Group PLLA-aligned/0.25% pDNM gel vs. PLLA-aligned/1% pDNM gel
[h]Group PLLA-aligned/0.25% pDNM gel vs. unstitched
[i]Group PLLA-aligned/1% pDNM gel vs. unstitched
[j]Group PLLA-aligned/1% pDNM gel vs. control
[k]Group PLLA-aligned/1% pDNM gel vs. PLLA-random
[l]Group PLLA-aligned vs. PLLA-random;

Table S5. SFI in Unstitched, Autograft, PLLA-random, PLLA-aligned, PLLA-aligned/0.25% pDNM gel, PLLA-aligned/1% pDNM gel, and Control groups

Time points (weeks)	Unstitched (mean ± SD)	Autograft (mean ± SD)	PLLA-random (mean ± SD)	PLLA-aligned (mean ± SD)	PLLA-aligned/0.25% pDNM gel (mean ± SD)	PLLA-aligned/1% pDNM gel (mean ± SD)	Control (mean ± SD)	$p < 0.002$
0 (Pre)	-9.021 ± 0.787	-8.883 ± 0.825	-8.843 ± 0.683	-8.594 ± 0.616	-8.900 ± 1.313	-9.009 ± 0.858	-8.628 ± 0.558	-
1	-105.278 ± 3.190	-100.146 ± 1.994	-104.820 ± 2.064	-105.515 ± 0.897	-101.809 ± 3.591	-104.681 ± 1.994	-8.945 ± 0.380	#
2	-97.047 ± 3.806	-72.065 ± 2.383	-91.908 ± 4.246	-88.078 ± 3.277	-81.863 ± 4.075	-89.075 ± 4.503	-8.922 ± 0.700	# †bd
6	-92.585 ± 2.395	-43.714 ± 3.362	-81.707 ± 3.396	-71.394 ± 6.321	-54.727 ± 4.554	-78.207 ± 5.535	- 8.745 ± 0.589	# †‡bce
8	-80.822 ±2.278	-25.477 ±3.862	-71.094 ±3.623	-59.949 ± 5.460	-44.949 ± 4.544	-66.761 ± 2.977	- 8.743 ± 0.244	# †‡abce

SD: standard deviation; $p < 0.002$

Statistically significant difference between
#: Group control vs. all the other groups
†: Group autograft vs. all the other groups
‡: Group unstitched vs. all the other groups
[a]Group PLLA-random vs. PLLA-aligned
[b]Group PLLA-random vs. PLLA-aligned/0.25% pDNM gel
[c]Group PLLA-aligned vs. PLLA-aligned/0.25% pDNM gel
[d]Group PLLA-aligned/0.25% pDNM gel vs. unstitched group
[e]Group PLLA-aligned/0.25% pDNM gel vs. PLLA-aligned/1% pDNM gel
